# Validation of the General and Sport Nutrition Knowledge Questionnaire (GeSNK) in Spanish Adolescents

**DOI:** 10.3390/nu14245324

**Published:** 2022-12-15

**Authors:** María Ángeles Manzano-Felipe, Celia Cruz-Cobo, María Ángeles Bernal-Jiménez, María José Santi-Cano

**Affiliations:** 1 Primary Health Care District of Bay of Cádiz-La Janda, 11006 Cádiz, Spain; 2 Research Group on Nutrition, Molecular, Pathophysiological and Social Issues, University of Cádiz, 11009 Cádiz, Spain; 3 Institute of Biomedical Research and Innovation of Cádiz (INiBICA), 11009 Cádiz, Spain; 4 Faculty of Nursing and Physiotherapy, University of Cádiz, 11009 Cádiz, Spain

**Keywords:** adolescents, nutritional knowledge, questionnaire, sports, young adults

## Abstract

The General and Sport Nutrition Knowledge Questionnaire (GeSNK) is an instrument that has been developed and validated to assess the level of nutrition knowledge in adolescents and young adults. The aim of the present study was to validate the GeSNK questionnaire in a group of Spanish adolescents in the framework of a Nutrition Education Programme in Secondary Schools in Andalusia, Spain. This cross-sectional questionnaire validation study was developed in two phases: translation-cultural adaptation and validation. A total of 305 adolescents aged 11 to 17 years, studying from the first to the third year of compulsory secondary education, participated on a voluntary basis. The GeSNK questionnaire consists of 62 items: 29 items for the General Nutrition section and 33 items for the Sports Nutrition section. Cronbach’s alpha coefficient for the complete questionnaire (GeSNK Total) was: 0.934; for the GeSNK General Nutrition section it was 0.918; and for the GeSNK Sports Nutrition section it was 0.856. The stability measured by the correlation coefficient for the General Nutrition section was 0.406 (*p* = 0.000); for the Sports Nutrition section it was 0.198 (*p* = 0.017); and for GeSNK Total the stability was 0.545 (*p* = 0.000). The questionnaire also demonstrated adequate construct validity. We therefore conclude that the Spanish version of the GeSNK questionnaire is a valid instrument to measure the level of knowledge in general nutrition and sports nutrition in adolescents.

## 1. Introduction

Nutritional knowledge, defined as the understanding of healthy nutrition concepts, can determine the eating behaviour and health habits of individuals from childhood onwards, along with other environmental, socioeconomic and cultural factors [1]. Such knowledge plays a role in the prevention of chronic diseases such as obesity. The prevalence of this disease is increasing at an alarming rate in developed countries [2].

Dietary behaviours are formed during early childhood [3,4,5] and are consolidated until adolescence. Therefore, adolescence may be a key stage in terms of nutrition education, as at this age more freedom is acquired in terms of food and drink choices outside the home [6].

Both to assess the level of knowledge in cross-sectional studies and to analyse the effectiveness of nutrition education programmes, questionnaires have been developed and validated to assess the level of knowledge about general nutrition [7,8,9], sports nutrition [10,11,12,13], specific foods or nutrients [14,15] or body weight control [16] applied to people of different ages from children to young people and adults.

The “General and Sport Nutrition Knowledge Questionnaire: GeSNK” is an instrument that has been developed and validated to assess the level of nutrition knowledge in Italian adolescents and young adults, obtaining good psychometric properties with an internal consistency value (Cronbach’s alpha) of 0.86 [17]. This questionnaire assesses both general nutrition knowledge and specific sports nutrition knowledge. 

Currently, to our knowledge, no tool is available that has been validated in Spain to assess nutritional knowledge in adolescents. Moreover, data on the nutritional knowledge of Spanish adolescents are very limited.

Therefore, the aim of the present study was to validate the General and Sport Nutrition Knowledge Questionnaire (GeSNK) in a group of Spanish adolescents by nurses responsible for school education in primary care in the framework of a Nutrition Education Programme in Secondary Schools of the Ministries of Health and Education in Andalusia, Spain.

## 2. Materials and Methods

### 2.1. Design

This cross-sectional questionnaire validation study was developed in two phases: translation-cultural adaptation and validation. 

### 2.2. Participants 

A total of 305 adolescents aged 11 to 17 years, studying from the first to the third year of secondary education in two public high schools in Cadiz, Andalusia, Spain, from October 2019 to January 2020, participated in the study on a voluntary basis. Sociodemographic data (date of birth, sex, academic year); anthropometric data (height and weight for the calculation of body mass index—kg/m^2^—); parents’ level of education; and parents’ employment status were collected through interviews with the participants and parents. The following levels of parental education were considered: no education, primary education, secondary education and university [18]. Parental employment status was classified as: active, unemployed and retired. All students received the GeSNK questionnaire.

### 2.3. GeSNK Questionnaire

The GeSNK questionnaire consists of initial questions on socio-demographic data and sports practice, and 62 items [17]; the General Nutrition section includes 29 items. The first eight items contain 43 questions on the macronutrient and micronutrient content of some foods and are answered with the options “high”, “low or “absent” and “don’t know”. The remaining questions are answered with “true”, “false”, “don’t know” or with only one true option. The maximum score for this section is 64 points. The Sports Nutrition section includes 33 items and is answered with “true”, “false”, “don’t know” or one true option. The maximum total score for the entire questionnaire is 97 points and the minimum score is 0. Scores are coded as +1 for a correct answer and 0 if participants select the wrong answer or the answer “I don’t know”. When adolescents fill in the GeSNK, a low level of knowledge is considered when the participants obtain a total score of less than 46 points (33rd percentile) (32 points for General Nutrition and 14 points for Nutrition and Sport). A total score between 46 and 58 points (33rd percentile to 66th percentile) is considered medium. A total score higher than 58 points (40 points in General Nutrition and 18 points in Nutrition and Sport) is classified as high knowledge. 

The students completed the self-administered questionnaire face-to-face during their class time. The principal investigator provided the questionnaire to the students and gave them verbal instructions on its completion and the importance of answering honestly.

### 2.4. Translation

Translation and cross-cultural adaptation of the original English version [17] to the Spanish version was carried out in order to obtain a semantic, conceptual and content-equivalent instrument. The method used was that of the translation and back-translation of the original version of the instrument with bilingual speakers. Two translations of the original version into Spanish were carried out. The translations were reviewed by two experts, a doctor and a nurse, with experience in nutrition and physical activity education programmes, who drafted the first version of the questionnaire. This version was back-translated into English by two bilingual persons. These versions were again reviewed by the experts and the final version of the questionnaire was decided upon. This version was pilot-tested on a group of 10 adolescents aged 11–17 years who were asked to explain the meaning in their own words and reported that they correctly understood the translation of the questionnaire. (The English and Spanish versions of the questionnaire are provided as Supplementary Materials S1 and S2.)

### 2.5. Validation

The psychometric properties of the instrument were assessed in terms of reliability and validity. 

Reliability was estimated by means of (a) internal consistency and (b) stability. Internal consistency was assessed through Cronbach’s alpha coefficient, considering a value of 0.7–0.8 as acceptable, 0.8–0.9 as good and >0.9 as excellent [19]. Stability was measured using the test–retest technique. After two weeks and under the same conditions, the questionnaire was delivered again to 145 adolescents and stability was assessed using Pearson’s correlation coefficient. The 2-week period was considered an acceptable time period for participants to exclude learning and memory effects [20]. 

To test construct validity, scores on the GeSNK questionnaire were compared between participants from the three secondary school grades, as it was expected that the level of knowledge would be higher in participants from higher grades. 

Feasibility was measured by the time required to complete the questionnaire.

### 2.6. Statistical Analysis

The statistical analysis was performed using SPSS 24.00 (IBM, New York, NY, USA). The sample size was estimated based on the number of participants with whom the initial validation was carried out [17]. Qualitative variables are shown as percentages and frequencies and continuous variables as mean and standard deviation. The Chi-squared/Fisher test was used for the comparison of proportions. The ANOVA test and post hoc tests were used for means comparison. Internal consistency was assessed through Cronbach’s alpha coefficient and stability was assessed using Pearson’s correlation coefficient. Statistical tests to estimate the reliability and validity of the questionnaire are described in the previous section (2.5. Validation). Values of *p* < 0.05 were considered statistically significant.

### 2.7. Ethical Considerations

The study was conducted in accordance with the General Ethical Principles established in the Declaration of Helsinki, updated in 2013 in Fortaleza (Brazil) for the conduct of research studies in human subjects, and complies with Law 14/2007 on Biomedical Research and the current European Data Protection Regulation. All the participants and their parents signed the informed consent form. The study obtained a favourable report from the Research Ethics Committee of Cadiz (Ref. CEIC_11_02_2019).

## 3. Results

The baseline characteristics of the 305 participants are shown in Table 1. The mean age was 13.5 ± 1.2 years and 49.2% (*n* = 150) were male. 

### 3.1. Reliability

Internal consistency was measured for the entire questionnaire and separately for the two sections of the GeSNK questionnaire: Section 1, GeSNK General Nutrition and Section 2, GeSNK Nutrition and Sport. Cronbach’s alpha coefficient for the complete questionnaire (GeSNK Total) was 0.934; for the section GeSNK General Nutrition it was 0.918; and for the section GeSNK Sports Nutrition it was 0.856. 

### 3.2. Stability

The stability measured by the correlation coefficient for the General Nutrition section was *r* = 0.406 (*p* = 0.000); for the Sports Nutrition section it was *r* = 0.198 (*p* = 0.017); and for GeSNK Total the stability was *r* = 0.545 (*p* = 0.000). 

### 3.3. Construct Validity

As expected, the participants showed higher scores on the level of knowledge in relation to the academic year. Adolescents in higher grades had significantly higher knowledge scores on both the GeSNK total score (*p* = 0.000) and the General Nutrition (*p* = 0.002) and Sports Nutrition (*p* = 0.000) sections (Table 2).

### 3.4. Questionnaire Scores

In terms of total scores and in the General Nutrition section, no significant gender differences were observed, although in the Sports Nutrition section, males scored significantly higher than females (Table 3).

A total of 59.1% of the participants showed a low level of knowledge, 26.5% a medium level of knowledge and 14.4% a high level of knowledge.

Regarding the initial questions of the questionnaire, the time spent exercising per week was 196.5 ± 141.9 min, which was similar in men and women (Table 3). 

In relation to the main sources of information on nutrition that the adolescents referred to, in first place they indicated nutrition education programmes at school (63.1%), followed by parents (59.1%), teachers (35.9%), television (26.8%), trainers (22.8%), the Internet (22.1%) and friends (7.0%). Only 4.4% of adolescents considered that they had no knowledge about nutrition.

There was a correlation between the total level of nutrition knowledge (GeSNK Total) and the academic level of the father (*r* = 0.148, *p* = 0.018), although there was no relationship between the level of knowledge and the occupation of the parents.

The time required to complete the questionnaire was 20–25 min.

## 4. Discussion

The present study validated the Spanish version of the GeSNK questionnaire, which showed its reliability by means of an internal consistency that was considered excellent, as well as by presenting an adequate construct validity. 

The demonstration of internal consistency is a key step in the psychometric validation of the questionnaire. The GeSNK questionnaire obtained good results in the total score (α = 0.934), the General Nutrition section (α = 0.918) and the Nutrition and Sport section (α = 0.856). The stability of the instrument was acceptable (*r* = 0.545, *p* = 0.000), although lower than that observed by Calella et al. [17], which could be attributed to the age of the participants in our study, which was lower than in the aforementioned study. 

Another instrument to measure the knowledge of general and sports nutrition in adolescents was recently validated in 264 Italian adolescents [8], consisting of a short questionnaire with 26 items that showed moderate internal consistency (α = 0.684) but good correlation over time (*r* = 0.977). 

Other recent studies, such as that of Rosi et al. [21], validated a nutrition knowledge questionnaire in 132 Italian university students (19–30 years old). The questionnaire revealed similar data to ours with a high overall internal consistency (α > 0.8) and good temporal stability with high correlation of the total score (*r* = 0.835). However, this questionnaire was longer than the one used in our study, with 90 items. Regarding the number of items, it has been shown that longer questionnaires seem to achieve lower completion rates and are, therefore, less practical in certain settings such as a school classroom [22].

The original version of the GeSNK questionnaire obtained Cronbach’s alpha values of 0.86 for total GeSNK, and 0.84 and 0.71 for the General and Sports Nutrition sections, respectively [17]. These authors classified a score below the 33rd percentile as a low knowledge level, which corresponded to 32 points for general nutrition, 14 points for nutrition and sport and 46 points for the total score. In our study, these scores were lower: 35 points in total GeSNK, 23 points in general nutrition and 12 in the sports section. These authors considered an average level of knowledge to be a score between the 33rd and 66th percentile, and performance above the 66th percentile was classified as high knowledge, which in their work was set at 58 points in total GeSNK, 40 points in general nutrition and 18 in nutrition and sport. In our research, these scores were also lower, with values of 48 points in GeSNK total, 31 points in general nutrition and 17 points in sports nutrition. These lower scores can be attributed to the fact that the mean age of the adolescent participants was lower than that of the Italian youth who constituted the validation sample in the study by Calella et al. [17] (13.5 ± 1.2 years vs. <16 years to >17 years).

The Turkish version of the GeSNK questionnaire was recently validated in adolescent athletes. The authors obtained good internal consistency (α = 0.884), although they reduced the number of items to 49 (25 in the General Nutrition section and 24 in the Sports Nutrition section), and the study was only conducted in males; therefore, the scores are not comparable [23].

Construct validity, which measures the ability of the questionnaire to distinguish between groups of individuals that are expected to be different, showed in our study that adolescents in upper secondary school had higher knowledge scores. These differences in knowledge scores were statistically significant for the total questionnaire, but also for the “General nutrition” and “Sports nutrition” sections, showing that our questionnaire can discriminate between adolescents with different levels of knowledge.

A correlation was also observed between the total level of knowledge of nutrition (GeSNK Total) and the academic level of the father. Although there was no relationship between the level of knowledge and the occupation/work of the parents, these data coincide with the results obtained by Calella et al. [17] in the validation of the GeSNK questionnaire in Italian adolescents in terms of the relationship with the parents’ work, but not with regard to the level of education of the parents, since in our study we did observe an association, as mentioned above.

Regarding the score on general nutrition knowledge, we found no significant differences between males and females; this is in line with the study by Brown et al. [24], who analysed food and nutrition knowledge among adolescents aged 13–19 years in the London metropolitan area and Canada, but differs from that reported in other recent studies, in which females scored higher than males [9,25,26,27]. However, in our research we observed that, in the Sports Nutrition section, male scores were statistically higher than female scores, which may suggest a greater interest of males in sports nutrition [28]. 

In relation to the main sources of information from which adolescents obtained their knowledge on nutrition, 26.4% of males pointed to their sports coach vs. 19.3% of females. 

The GNKQ (General Nutrition Knowledge Questionnaire) [29], developed in the 1990s for the UK population and updated in 2016 [30], has been widely used in research and validated in various populations. Recent studies, such as Putnoky et al. [26], validated it in a population of 411 Romanian athletes, obtaining an overall internal reliability of 0.878 and an external reliability of >0.880 in all sections and globally. On the other hand, Gao et al. [9] validated this tool in 278 Chinese adults, obtaining an overall internal consistency coefficient of 0.885 and a test–retest reliability of 0.769. It was also validated in an Arab population, with a sample of 805 university students, showing an internal reliability (Cronbach’s α = 0.91) and an external reliability of 0.350, and an intraclass correlation coefficient of 0.84. On the other hand, Thompson et al. [31] validated it in Australian university students, comparing nutrition students with engineering students and obtaining statistically significant differences between the two groups. The internal reliability of the questionnaire was high (α = 0.92), as was test–retest reliability (*r* = 0.96). However, all these studies were conducted in an adult population. Furthermore, this tool addresses general nutrition knowledge but not sports nutrition knowledge, which is of increasing interest among young people and adolescents, and this is an area where there are many knowledge gaps. Recent studies point to the increasing use of energy supplements and snacks, such as unhealthy energy bars and soft drinks, by adolescent athletes [32,33]. The GeSNK questionnaire, however, targets adolescents as well as young adults, the general population and athletes.

Vázquez-Espino et al. [34] developed a 59-item questionnaire on nutrition knowledge for young and adult athletes (NUKYA) in Spain to assess the main bases of nutrition knowledge specifically in team sports. The authors found statistically significant differences when comparing the group of participants with nutrition knowledge (final year nutrition students) with the rest of the groups without nutrition knowledge (philosophy students, athletes and non-athletes), with the test–retest reliability of the questionnaire (*r* = 0.895) and high internal consistency (α = 0.849). Tam et al. [12] also validated a sports nutrition knowledge tool in 255 athletes through the questionnaire (PEAKS-NQ) with 94 items. The authors obtained a Cronbach’s α of 0.86 and good content validity (0.88).

In our study we were unable to analyse concurrent validity, as we currently do not have other nutrition knowledge questionnaires validated in Spanish for this age group. In this regard, a recent systematic review that analysed the instruments used to measure nutritional knowledge in publications on educational interventions in the school setting in adolescents concluded that there was a lack of description of these instruments, as well as their psychometric properties. They also stated that this omission makes it difficult to choose appropriate instruments for use in such interventions [35].

As we have seen, there are few instruments that measure the knowledge of sports nutrition, as well as general nutritional knowledge, in both adolescents and adults; thus, the GeSNK questionnaire is a complete, valid and reliable instrument addressing both general nutrition knowledge and sports nutrition knowledge.

One of the strengths of this study is the large sample of adolescents who participated in the completion of the questionnaire for its validation. To our knowledge, this study is the first to provide a validated questionnaire of these characteristics for use in Spanish adolescents. The questionnaire was completed in paper format, not online, so no one was excluded if they did not have access to the Internet or a computer. 

### Limitations

There are some limitations that may affect the generalisability of these results, as the study was conducted in two secondary schools in the province of Cadiz, Andalusia, Spain, so it is possible that the sample does not reflect the educational level of adolescents in general. There was no control group, however, to analyse construct validity. The scores obtained in the GeSNK questionnaire were compared between the participants of the three secondary school grades, as it could be expected that the level of knowledge would be higher in the participants of higher grades; therefore, this limitation seems unimportant. The possibility for participants to search for the correct answer or guess the correct answer is also a limitation. Therefore, in order to reduce guessing, all questions had a “don’t know” option, and the participants were instructed to mark “don’t know” instead of guessing the answer, and as the questionnaire was conducted in a supervised school classroom, there was also no option to search for the answer via electronic means (mobile phone, computer).

## 5. Conclusions

Finally, the Spanish version of the GeSNK questionnaire is a valid instrument to measure the level of knowledge of general nutrition and sports nutrition in adolescents. In addition, this questionnaire can be useful to evaluate nutrition education programmes and to detect gaps in nutrition knowledge that require further attention.

Validated tools to assess nutritional knowledge among adolescents are needed to investigate the relationship between dietary habits and nutritional knowledge in this population.

## Figures and Tables

**Table 1:** General characteristics of the participants.

**Sex % (n)**	
Male	49.2% (150)
Female	50.8% (155)
**Age**	13.5 ± 1.2
**Academic year**	
Academic year 1st	34.4% (105)
Academic year 2nd	42.3% (129)
Academic year 3rd	23.3% (71)
**Father’s academic level**	
No studies	8.8% (23)
Primary education	47.7% (124)
Secondary	33.8% (88)
University studies	9.6% (25)
**Mother’s academic level**	
No studies	4.5% (12)
Primary education	45.4% (122)
High school	33.8% (91)
University	16.4% (44)
**Father’s occupation**	
Active	84.2% (218)
Unemployed	10.0% (26)
Retired	5.8% (15)
**Mother’s occupation**	
Active	61.0% (164)
Unemployed	36.4% (98)
Retired	2.6% (7)

**Table 2:** GeSNK knowledge level score of the participants in the three secondary school years.

	Total	1st Year	2nd Year	3rd Year	*p*
Total-GeSNK (points)	42.6 ± 6.7	41.9 ± 5.4	41.5 ± 7.9	45.8 ± 4.8	0.000
GeSNK General Nutrition (points)	27.8 ± 4.7	27.0 ± 3.9	27.4 ± 5.6	29.6 ± 3.5	0.002
GeSNK Sport Nutrition (points)	14.8 ± 3.2	14.8 ± 3.0	14.0 ± 3.5	16.1 ± 2.6	0.000

ANOVA post hoc: Total—GeSNK, 1st year vs. 3rd year, *p* = 0.001; 2nd vs. 3rd year, *p*= 0.000. GeSNK General Nutrition, 1st year vs. 3rd year, *p* = 0.002; 2nd vs. 3rd, *p* = 0.007. GeSNK Sport Nutrition, 1st year vs. 3rd year, *p* = 0.027; 2nd year vs. 3rd year, *p* = 0.000.

**Table 3:** GeSNK knowledge score in relation to gender.

	Total	Male	Female	*p*
**GeSNK**				
Total GeSNK (points)	41.6 ± 14.6	41.6 ± 15.7	41.6 ± 13.4	0.980
GeSNK General Nutrition (points)	27.4 ± 10.3	26.8 ± 10.9	27.9 ± 9.8	0.349
GeSNK Sport Nutrition (points)	14.3 ± 5.7	15.03 ± 6.0	13.6 ± 5.2	0.040
Total GeSNK Low level % (*n*)	59.1% (176)	60.1% (89)	58.0% (87)	0.133
Total GeSNK Medium level % (*n*)	26.5% (79)	22.3% (33)	30.7% (46)	
Total GeSNK High level % (*n*)	14.4% (43)	17.6% (26)	11.3% (17)	
**Exercise** (min/week)	196.5 ± 141.9	200.5 ± 147	192.6 ± 136	0.632
**Origin of knowledge in Nutrition**				
School-based nutrition education programmes	63.1% (188)	61.5% (91)	64.7% (97)	0.631
Nutrition education programmes elsewhere	19.1% (57)	19.6% (29)	18.7% (28)	0.883
Teachers	35.9% (107)	35.8% (53)	36.0% (54)	0.535
Parents	59.1% (176)	54.1% (80)	64.0% (96)	0.099
Coaches	22.8% (68)	26.4% (39)	19.3% (29)	0.168
Television	26.8% (80)	27.0% (40)	26.7% (40)	1.000
Internet	22.1% (66)	23.6% (35)	20.7% (31)	0.578
Friends	7.0% (21)	6.1% (9)	8.0% (12)	0.652
I have no knowledge of healthy eating	4.4% (13)	4.1% (6)	4.7% (7)	1.000
Other	3.0% (9)	0.7% (1)	5.3% (8)	0.036

## Data Availability

The data supporting the current findings can be found upon request to the corresponding author in the repository of scientific data from the local university.

## Supplementary Materials

The following supporting information can be downloaded at: https://www.mdpi.com/article/10.3390/nu14245324/s1, Material S1: English version of the GeSNK questionnaire; Material S2: Spanish version of the GeSNK questionnaire.
