# Supplementary material for: Validation of the General and Sport Nutrition Knowledge Questionnaire (GeSNK) in Spanish Adolescents
Source: Nutrients. 2022 Dec 15;14(24):5324. doi: 10.3390/nu14245324 (PMC9786199; doi:10.3390/nu14245324)
Supplement: Supplementary file 1 [file nutrients-14-05324-s001.zip › nutrients-2081961-supplementary.pdf]

S1. GeNSK- General and Sport Nutrition Knowledge Questionnaire

The purpose of this questionnaire is to identify myths and false nutritional beliefs in order to improve nutritional education programs

Name/Surname:
Gender: Male ☐ Female ☐
Date of Birth:
Weight: Height:
Employment father/mother:

Level of education father/mother:
Do you practice any Sport? YES☐ NO☐
Sport:
How many times a week:
Average length: *(minutes per day)***:**

My knowledge about healthy eating are driven by: (you can choose more than one answer)

Nutritional education programs at school	☐	*From what I watch on the TV*	☐
Nutritional education programs in other places	☐	*From the web*	☐
From my teachers	☐	*From my friends*	☐
From my parents	☐	*I have no knowledge about to healthy eating*	☐
From my coaches	☐	*Anything else………………………………………………….*	

SECTION 1: GENERAL NUTRITION

Following are the questions refer to the nutritional composition of some foods. Choose the answer with an X.

1. **The Carbohydrate content of such foods is:**

	High	Low or absent	I do not know
Boiled ham	☐	* ☐	☐
White bread	* ☐	☐	☐
Tomato	☐	* ☐	☐
Apple	☐	* ☐	☐
Ricotta	☐	* ☐	☐
Breakfast cereals	* ☐	☐	☐

2. **The Protein content of such foods is:**

	High	Low or absent	I do not know
Chicken meat	* ☐	☐	☐
Dried beans	* ☐	☐	☐
Pear	☐	* ☐	☐
Rice	☐	* ☐	☐
Codfish	* ☐	☐	☐
Parmesan	* ☐	☐	☐
Chocolate	☐	* ☐	☐

3. **The Fat content of such foods is:**

	High	Low or absent	I do not know
Salami	* ☐	☐	☐
Mayonnaise	* ☐	☐	☐
Dried chickpeas	☐	* ☐	☐
Pasta	☐	* ☐	☐
Butter	* ☐	☐	☐
Jam	☐	* ☐	☐

4. **The Fiber content of such foods is:**

	High	Low or absent	I do not know
Honey	☐	* ☐	☐
Brown bread	* ☐	☐	☐
Chicken broth	☐	* ☐	☐
Potatoes	☐	* ☐	☐
Pear	* ☐	☐	☐
White bread	☐	* ☐	☐

5. **The <u>Salt</u> content of such foods is:**

	High	Low or absent	I do not know
White bread	° ☐	☐	☐
Courgettes	☐	* ☐	☐
Canned peas	* ☐	☐	☐
Canned tuna	* ☐	☐	☐
Frozen peas	☐	* ☐	☐

6. **The <u>Calcium</u> content of such food is:**

	High	Low or absent	I do not know
Turkey breast	☐	* ☐	☐
Peas	☐	* ☐	☐
Walnut	* ☐	☐	☐
Olive oil	☐	* ☐	☐
Brown bread	* ☐	☐	☐

7. **The <u>Iron</u> content of such foods is:**

	High	Low or absent	I do not know
Calf meat	* ☐	☐	☐
Apple	☐	* ☐	☐
Honey	☐	* ☐	☐
Sea bass	* ☐	☐	☐

8. **The <u>Potassium</u> content of such foods is:**

	High	Low or absent	I do not know
Pasta	☐	* ☐	☐
Dried lentils	* ☐	☐	☐
Olive oil	☐	* ☐	☐
Honey	☐	* ☐	☐

Are these claims true or false? (only one answer is possible)

**9**	*The egg white is high in cholesterol*		
	**TRUE** ☐	***FALSE** ☐	**I DO NOT KNOW** ☐
**10**	*The high-fat meals are ever high in cholesterol*		
	**TRUE** ☐	***FALSE** ☐	**I DO NOT KNOW** ☐
**11**	*The olive oil is high in monounsaturated fat*		
	***TRUE** ☐	**FALSE** ☐	**I DO NOT KNOW** ☐
**12**	*The dried fruit is a good source of essential fatty acids*		
	***TRUE** ☐	**FALSE** ☐	**I DO NOT KNOW** ☐
**13**	*The ripened cheese are saltier than the fresh one*		
	***TRUE** ☐	**FALSE** ☐	**I DO NOT KNOW** ☐
**14**	*An high-energy food is exclusively a fat food*		
	**TRUE** ☐	***FALSE** ☐	**I DO NOT KNOW** ☐
**15**	*The brown bread is richer in fiber than the white one*		
	***TRUE** ☐	**FALSE** ☐	**I DO NOT KNOW** ☐
**16**	*Bran is the outer part of the grain kernel that is very high in fiber*		
	***TRUE** ☐	**FALSE** ☐	**I DO NOT KNOW** ☐
**17**	*Tinned pulses are saltier than the dry one*		
	***TRUE** ☐	**FALSE** ☐	**I DO NOT KNOW** ☐
**18**	*Omega-3 and omega-6 are particular fatty acids*		
	***TRUE** ☐	**FALSE** ☐	**I DO NOT KNOW** ☐
**19**	*Our body creates vitamin D from direct sunlight on our skin when we are outdoors*		
	***TRUE** ☐	**FALSE** ☐	**I DO NOT KNOW** ☐
**20**	*The iron in meat are more easily absorbed than the same mineral found in vegetables*		
	***TRUE** ☐	**FALSE** ☐	**I DO NOT KNOW** ☐
**21**	*A variety of foods contain a natural amount of sodium*		
	***TRUE** ☐	**FALSE** ☐	**I DO NOT KNOW** ☐
**22**	*Dairy are a good iron source*		
	**TRUE** ☐	***FALSE** ☐	**I DO NOT KNOW** ☐
**23**	*Carrots are a good source of vitamin A*		
	***TRUE** ☐	**FALSE** ☐	**I DO NOT KNOW** ☐

24. The glycemic index food: (only one answer is possible)

indicates the foods carbohydrates content		☐
indicates the food's effect on a person's blood glucose	*	☐
indicates the food's effect on a person's blood glucose after the ingestion of protein		☐
indicates the foods energy density		☐

Are these claims true or false? (only one answer is possible)

**25**	*An unbalanced diet is the only risk factor for the development of cardiovascular disease*		
	**TRUE** ☐	***FALSE** ☐	**I DO NOT KNOW** ☐
**26**	*In the obesity the diet play an important role, physical activity no*		
	**TRUE** ☐	***FALSE** ☐	**I DO NOT KNOW** ☐
**27**	*A low calcium and vitamin D intake during life, associated with a lack of physical activity may increase fracture risk*		
	***TRUE** ☐	**FALSE** ☐	**I DO NOT KNOW** ☐
**28**	*The fiber helps to ease constipation*		
	***TRUE** ☐	**FALSE** ☐	**I DO NOT KNOW** ☐
**29**	*To obtain a healthy weight loss the carbohydrates must not be removed from the diet*		
	***TRUE** ☐	**FALSE** ☐	**I DO NOT KNOW** ☐

SECTION 2: SPORT NUTRITION

Are these claims true or false? (only one answer is possible)

**30**	*To eat carbohydrate is no good for an athlete*		
	**TRUE** ☐	***FALSE** ☐	**I DO NOT KNOW** ☐
**31**	*B-group vitamins play an important role in muscle metabolism*		
	***TRUE** ☐	**FALSE** ☐	**I DO NOT KNOW** ☐
**32**	*Athletes must reduce the fat intake to a minimum*		
	**TRUE** ☐	***FALSE** ☐	**I DO NOT KNOW** ☐
**33**	*Consuming carbohydrates after 5 pm can enhance performance*		
	**TRUE** ☐	***FALSE** ☐	**I DO NOT KNOW** ☐
**34**	*Eating more protein will make muscles bigger*		
	**TRUE** ☐	***FALSE** ☐	**I DO NOT KNOW** ☐
**35**	*For a sporty person, the daily intake should not include more than 15% of fats*		
	**TRUE** ☐	***FALSE** ☐	**I DO NOT KNOW** ☐
**36**	*Athletes can eat whatever they want because they have a fast metabolism*		
	**TRUE** ☐	***FALSE** ☐	**I DO NOT KNOW** ☐
**37**	*Physical exercise is the main factor improving muscular strength*		
	***TRUE** ☐	**FALSE** ☐	**I DO NOT KNOW** ☐
**38**	*An excessive dietary protein intake can lead to liver and kidney damage*		
	***TRUE** ☐	**FALSE** ☐	**I DO NOT KNOW** ☐
**39**	*An athlete must have a meal at the end of the training session*		
	***TRUE** ☐	**FALSE** ☐	**I DO NOT KNOW** ☐
**40**	*To reduce pasta, potato and bread intake during the training period is needed*		
	**TRUE** ☐	***FALSE** ☐	**I DO NOT KNOW** ☐
**41**	*A man and woman of the same age, practicing the same sport have the same energy requirements*		
	**TRUE** ☐	***FALSE** ☐	**I DO NOT KNOW** ☐
**42**	*It is advisable for an athlete eating a low glycemic index meal but rich in carbohydrates, 1-2 hours after training*		
	***TRUE** ☐	**FALSE** ☐	**I DO NOT KNOW** ☐
**43**	*Athletes practicing extensive training have double protein requirement than the general population*		
	***TRUE** ☐	**FALSE** ☐	**I DO NOT KNOW** ☐
**44**	*Drinking fluids before, during and after a competition is needed*		
	***TRUE** ☐	**FALSE** ☐	**I DO NOT KNOW** ☐
**45**	*Coaches must not allow drinking fluid during a training*		
	**TRUE** ☐	***FALSE** ☐	**I DO NOT KNOW** ☐
**46**	*The best advice for athletes is to drink when they are thirsty*		
	**TRUE** ☐	***FALSE** ☐	**I DO NOT KNOW** ☐
**47**	*For an athlete, cold water quenches thirst better*		
	**TRUE** ☐	***FALSE** ☐	**I DO NOT KNOW** ☐
**48**	*Athletes can use ice cube to quench their thirst during training*		
	**TRUE** ☐	***FALSE** ☐	**I DO NOT KNOW** ☐
**49**	*A great deal to support performance in athletes is water*		
	***TRUE** ☐	**FALSE** ☐	**I DO NOT KNOW** ☐

**50**	*Sports drinks and energy drinks are the same things*		
	**TRUE** ☐	***FALSE** ☐	**I DO NOT KNOW** ☐
**51**	*Sports drinks contain minerals*		
	***TRUE** ☐	**FALSE** ☐	**I DO NOT KNOW** ☐

52.What is the most appropriate beverage after two hours of training?

Energy drink	☐
Sports drink	*☐
Fruit juice	☐
Cola	☐
I do not know	☐

Are these claims true or false? (only one answer is possible)

**53**	*For athletes, supplements can be used as valid meals replacement*		
	**TRUE** ☐	***FALSE** ☐	**I DO NOT KNOW** ☐
**54**	*For athletes, it is impossible to reach the iron requirement they need supplements*		
	**TRUE** ☐	***FALSE** ☐	**I DO NOT KNOW** ☐
**55**	*Sports drinks contain caffeine*		
	**TRUE** ☐	***FALSE** ☐	**I DO NOT KNOW** ☐
**56**	*If athletes are not able to fulfill their proteins requirement, they need to take amino acids supplements*		
	***TRUE** ☐	**FALSE** ☐	**I DO NOT KNOW** ☐
**57**	*Supplements are necessary both in professional and recreational sports*		
	**TRUE** ☐	***FALSE** ☐	**I DO NOT KNOW** ☐
**58**	*Food supplements are safe so it is right to use them without any expert advice*		
	**TRUE** ☐	***FALSE** ☐	**I DO NOT KNOW** ☐
**59**	*In strength sports, vitamin c supplements are ever required*		
	**TRUE** ☐	***FALSE** ☐	**I DO NOT KNOW** ☐
**60**	*Unseasoned meals are the best choice for muscle building and toning*		
	**TRUE** ☐	***FALSE** ☐	**I DO NOT KNOW** ☐
**61**	*Meat and egg white contain protein, other foods not. This is the foundation of the athlete's nutrition*		
	**TRUE** ☐	***FALSE** ☐	**I DO NOT KNOW** ☐
**62**	*Athletes can eat "light" products at will*		
	**TRUE** ☐	***FALSE** ☐	**I DO NOT KNOW** ☐

S2. GeNSK- Cuestionario de Conocimientos sobre Nutrición General y Deportiva

El objetivo de este cuestionario es identificar mitos y falsas creencias nutricionales para mejorar los programas de educación nutricional

Nombre/Apellido:
Género: **Mujer** ☐ **Hombre** ☐
Fecha de nacimiento:
Peso: **Altura:**
Empleo padre/madre:

Nivel de educación padre/madre:
¿Practica algún deporte? **SÍ** ☐ **NO** ☐
Deporte:
¿Cuántas veces a la semana:
Duración media: *(minutos al día):*

Mis conocimientos sobre la alimentación saludable están impulsados por: (puede elegir más de una respuesta)

Programas de educación nutricional en la escuela	☐	*Por lo que veo en la televisión*	☐
Programas de educación nutricional en otros lugares	☐	*De internet*	☐
De mis profesores	☐	*De mis amigos*	☐
De mis padres	☐	*No tengo conocimientos sobre nutrición saludable*	☐
De mis entrenadores	☐	*De:*	

SECCIÓN 1: NUTRICIÓN GENERAL

Las siguientes preguntas se refieren a la composición nutricional de algunos alimentos. Elige la respuesta con una X.

1. **El contenido de <u>carbohidratos</u> de estos alimentos es:**

	Alto	Bajo o ausente	No lo sé
Jamón cocido	☐	* ☐	☐
Pan blanco	* ☐	☐	☐
Tomate	☐	* ☐	☐
Manzana	☐	* ☐	☐
Requesón	☐	* ☐	☐
Cereales desayuno	* ☐	☐	☐

2. **El contenido en <u>proteínas</u> de estos alimentos es:**

	Alto	Bajo o ausente	No lo sé
Carne de pollo	* ☐	☐	☐
Judias	* ☐	☐	☐
Pera	☐	* ☐	☐
Arroz	☐	* ☐	☐
Bacalao	* ☐	☐	☐
Parmesano	* ☐	☐	☐
Chocolate	☐	* ☐	☐

3. **El contenido de <u>grasa</u> de estos alimentos es:**

	Alto	Bajo o ausente	No lo sé
Salami	* ☐	☐	☐
Mayonesa	* ☐	☐	☐
Garbanzos secos	☐	* ☐	☐
Pasta	☐	* ☐	☐
Manteqilla	* ☐	☐	☐
Jamón	☐	* ☐	☐

4. **El contenido de <u>fibra</u> de estos alimentos es:**

	Alto	Bajo o ausente	No lo sé
Miel	☐	* ☐	☐
Pan integral	* ☐	☐	☐
Caldo de pollo	☐	* ☐	☐
Patatas	☐	* ☐	☐
Pera	* ☐	☐	☐
Pan blanco	☐	* ☐	☐

5. **El contenido de <u>sal</u> de estos alimentos es:**

	Alto	Bajo o ausente	No lo sé
Pan blanco	° ☐	☐	☐
Calabacines	☐	* ☐	☐
Guisantes en lata	* ☐	☐	☐
Atún en lata	* ☐	☐	☐
Guisantes congelados	☐	* ☐	☐

6. **El contenido de <u>calcio</u> de estos alimentos es:**

	Alto	Bajo o ausente	No lo sé
Pechuga de pavo	☐	* ☐	☐
Guisantes	☐	* ☐	☐
Nueces	* ☐	☐	☐
Aceite de oliva	☐	* ☐	☐
Pan integral	* ☐	☐	☐

7. **El contenido de <u>hierro</u> de estos alimentos es:**

	Alto	Bajo o ausente	No lo sé
Carne de ternera	* ☐	☐	☐
Manzana	☐	* ☐	☐
Miel	☐	* ☐	☐
Lubina	* ☐	☐	☐

8. **El contenido de <u>potasio</u> de estos alimentos es:**

	Alto	Bajo o ausente	No lo sé
Pasta	☐	* ☐	☐
Lentejas secas	* ☐	☐	☐
Aceite de oliva	☐	* ☐	☐
Miel	☐	* ☐	☐

¿Son estas afirmaciones verdaderas o falsas? (sólo es posible una respuesta)

**9**	*La clara del huevo tiene un alto contenido de colesterol*		
	**VERDADERO** ☐	***FALSO** ☐	**NO LO SÉ** ☐
**10**	*Las comidas con alto contenido en grasa son siempre altas en colesterol*		
	**VERDADERO** ☐	***FALSO** ☐	**NO LO SÉ** ☐
**11**	*El aceite de oliva es rico en grasas monoinsaturadas*		
	*** VERDADERO** ☐	**FALSO** ☐	**NO LO SÉ** ☐
**12**	*Los frutos secos son una buena fuente de ácidos grasos esenciales*		
	*** VERDADERO** ☐	**FALSO** ☐	**NO LO SÉ** ☐
**13**	*Los quesos maduros son más salados que los frescos*		
	*** VERDADERO** ☐	**FALSO** ☐	**NO LO SÉ** ☐
**14**	*Un alimento muy energético es exclusivamente un alimento graso*		
	**VERDADERO** ☐	***FALSO** ☐	**NO LO SÉ** ☐
**15**	*El pan integral es más rico en fibra que el blanco*		
	*** VERDADERO** ☐	**FALSO** ☐	**NO LO SÉ** ☐
**16**	*El salvado es la parte exterior del grano que tiene un alto contenido en fibra*		
	*** VERDADERO** ☐	**FALSO** ☐	**NO LO SÉ** ☐
**17**	*Las legumbres en conserva son más saladas que las secas*		
	*** VERDADERO** ☐	**FALSO** ☐	**NO LO SÉ** ☐
**18**	*El omega-3 y omega-6 son ácidos grasos particulares*		
	*** VERDADERO** ☐	**FALSO** ☐	**NO LO SÉ** ☐
**19**	*Nuestro cuerpo crea vitamina D a partir de la luz solar directa sobre nuestra piel cuando estamos al aire libre*		
	*** VERDADERO** ☐	**FALSO** ☐	**NO LO SÉ** ☐
**20**	*El hierro de la carne se absorbe más fácilmente que el mismo mineral que se encuentra en las verduras*		
	*** VERDADERO** ☐	**FALSO** ☐	**NO LO SÉ** ☐
**21**	*Diversos alimentos contienen una cantidad natural de sodio*		
	*** VERDADERO** ☐	**FALSO** ☐	**NO LO SÉ** ☐
**22**	*Los lácteos son una buena fuente de hierro*		
	**VERDADERO** ☐	***FALSO** ☐	**NO LO SÉ** ☐
**23**	*Las zanahorias son una buena fuente de vitamina A*		
	*** VERDADERO** ☐	**FALSO** ☐	**NO LO SÉ** ☐

24. El índice glucémico de un alimento: (sólo es posible una respuesta)

Indica el contenido de carbohidratos de los alimentos		☐
Indica el efecto de los alimentos sobre la glucosa en sangre de una persona	*	☐
Indica el efecto de los alimentos sobre la glucosa en sangre de una persona tras la ingestión de proteínas		☐
Indica la densidad energética de los alimentos		☐

¿Son estas afirmaciones verdaderas o falsas? (sólo es posible una respuesta)

**25**	*Una dieta desequilibrada es el único factor de riesgo para el desarrollo de enfermedades cardiovasculares*		
	**VERDADERO** ☐	***FALSO** ☐	**NO LO SÉ** ☐
**26**	*En la obesidad la dieta juega un papel importante, la actividad física no*		
	**VERDADERO** ☐	***FALSO** ☐	**NO LO SÉ** ☐
**27**	*Una baja ingesta de calcio y vitamina D durante la vida, asociada a la falta de actividad física, puede aumentar el riesgo de fracturas*		
	*** VERDADERO** ☐	**FALSO** ☐	**NO LO SÉ** ☐
**28**	*La fibra ayuda a aliviar el estreñimiento*		
	*** VERDADERO** ☐	**FALSO** ☐	**NO LO SÉ** ☐
**29**	*Para conseguir una pérdida de peso saludable no se deben eliminar los carbohidratos de la dieta*		
	*** VERDADERO** ☐	**FALSO** ☐	**NO LO SÉ** ☐

SECTION 2: NUTRICIÓN DEPORTIVA

¿Son estas afirmaciones verdaderas o falsas? (sólo es posible una respuesta)

**30**	*Comer carbohidratos no es bueno para un atleta*		
	**VERDADERO** ☐	***FALSO** ☐	**NO LO SÉ** ☐
**31**	*Las vitaminas del grupo B desempeñan un papel importante en el metabolismo muscular*		
	***VERDADERO** ☐	**FALSO** ☐	**NO LO SÉ** ☐
**32**	*Los deportistas deben reducir la ingesta de grasa al mínimo*		
	**VERDADERO** ☐	***FALSO** ☐	**NO LO SÉ** ☐
**33**	*Consumir hidratos de carbono después de las 17 h puede mejorar el rendimiento*		
	**VERDADERO** ☐	***FALSO** ☐	**NO LO SÉ** ☐
**34**	*Comer más proteínas hará que los músculos sean más grandes*		
	**VERDADERO** ☐	***FALSO** ☐	**NO LO SÉ** ☐
**35**	*Para una persona deportista, la ingesta diaria no debe incluir más del 15% de grasas*		
	**VERDADERO** ☐	***FALSO** ☐	**NO LO SÉ** ☐
**36**	*Los deportistas pueden comer lo que quieran porque tienen un metabolismo rápido*		
	**VERDADERO** ☐	***FALSO** ☐	**NO LO SÉ** ☐
**37**	*El ejercicio físico es el principal factor de mejora de la fuerza muscular*		
	***VERDADERO** ☐	**FALSO** ☐	**NO LO SÉ** ☐
**38**	*Un consume excesivo de proteínas en la dieta puede provocar daños en el hígado y los riñones*		
	***VERDADERO** ☐	**FALSO** ☐	**NO LO SÉ** ☐
**39**	*El deportista debe comer al final de la sesión de entrenamiento*		
	***VERDADERO** ☐	**FALSO** ☐	**NO LO SÉ** ☐
**40**	*Es necesario reducir la ingesta de pasta, patatas y pan durante el periodo de entrenamiento*		
	**VERDADERO** ☐	***FALSO** ☐	**NO LO SÉ** ☐
**41**	*Un hombre y una mujer de la misma edad que practican el mismo deporte tienen las mismas necesidades energéticas*		
	**VERDADERO** ☐	***FALSO** ☐	**NO LO SÉ** ☐
**42**	*Es aconsejable que el deportista tome una comida de bajo índice glucémico pero rica en hidratos de carbono, 1-2h después del entrenamiento*		
	**VERDADERO** ☐	**FALSO** ☐	**NO LO SÉ** ☐
**43**	*Los deportistas que practican un entrenamiento exhaustivo tienen el doble de necesidades proteicas que la población general*		
	**VERDADERO** ☐	**FALSO** ☐	**NO LO SÉ** ☐
**44**	*Es necesario beber líquidos antes, durante y después de una competición*		
	**VERDADERO** ☐	**FALSO** ☐	**NO LO SÉ** ☐
**45**	*Los entrenadores no deben permitir que se beba líquido durante un entrenamiento*		
	**VERDADERO** ☐	***FALSO** ☐	**NO LO SÉ** ☐
**46**	*El mejor consejo para los deportistas es que beban cuando tengan sed*		
	**VERDADERO** ☐	***FALSO** ☐	**NO LO SÉ** ☐
**47**	*Para un deportista, el agua fría calma mejor la sed*		
	**VERDADERO** ☐	***FALSO** ☐	**NO LO SÉ** ☐
**48**	*Los deportistas pueden utilizar cubitos de hielo para calmar la sed durante el entrenamiento*		
	**VERDADERO** ☐	***FALSO** ☐	**NO LO SÉ** ☐
**49**	*El agua es una gran ayuda para el rendimiento de los deportistas*		
	**VERDADERO** ☐	**FALSO** ☐	**NO LO SÉ** ☐

**50**	*Las bebidas deportivas y las energéticas son lo mismo*		
	**VERDADERO** ☐	***FALSO** ☐	**NO LO SÉ** ☐
**51**	*Las bebidas deportivas contienen minerales*		
	***VERDADERO** ☐	**FALSO** ☐	**NO LO SÉ** ☐

52. ¿Cuál es la bebida más adecuada después de dos horas de formación?

Bebida energética	☐
Bebida deportiva	*☐
Zumo de frutas	☐
Cola	☐
No sé	☐

¿Son estas afirmaciones verdaderas o falsas? (sólo es posible una respuesta)

**53**	*Para los deportistas, los suplementos pueden utilizarse como sustituto válido de las comidas*		
	**VERDADERO** ☐	***FALSO** ☐	**NO LO SÉ** ☐
**54**	*Para los deportistas, es imposible alcanzar los requerimientos de hierro, necesitan suplementos*		
	**VERDADERO** ☐	***FALSO** ☐	**NO LO SÉ** ☐
**55**	*Las bebidas deportivas contienen cafeína*		
	**VERDADERO** ☐	***FALSO** ☐	**NO LO SÉ** ☐
**56**	*Si los deportistas no son capaces de satisfacer sus necesidades de proteínas, necesitan tomar suplementos de aminoácidos*		
	***VERDADERO** ☐	**FALSO** ☐	**NO LO SÉ** ☐
**57**	*Los suplementos son necesarios tanto en el deporte profesional como en el recreativo*		
	**VERDADERO** ☐	***FALSO** ☐	**NO LO SÉ** ☐
**58**	*Los complementos alimenticios son seguros, por lo que es correcto utilizarlos sin el asesoramiento de un experto*		
	**VERDADERO** ☐	***FALSO** ☐	**NO LO SÉ** ☐
**59**	*En los deportes de fuerza, los suplementos de vitamina C son siempre necesarios*		
	**VERDADERO** ☐	***FALSO** ☐	**NO LO SÉ** ☐
**60**	*Las comidas sin condimentar son la mejor opción para la construcción de músculos y la tonificación*		
	**VERDADERO** ☐	***FALSO** ☐	**NO LO SÉ** ☐
**61**	*La carne y la clara de huevo contienen proteínas, los demás alimentos no. Esta es la base de la nutrición del deportista*		
	**VERDADERO** ☐	***FALSO** ☐	**NO LO SÉ** ☐
**62**	*Los deportistas pueden comer productos "light" a su gusto*		
	**VERDADERO** ☐	***FALSO** ☐	**NO LO SÉ** ☐
